# Supplementary material for: Acceptance and compliance with micronutrient powder (MNP) among children aged 6–23 months in northern Nigeria
Source: PLOS Glob Public Health. 2022 Oct 17;2(10):e0000961. doi: 10.1371/journal.pgph.0000961 (PMC10022258; doi:10.1371/journal.pgph.0000961)
Supplement: S1 Dataset — (ZIP) [file pgph.0000961.s008.zip › S8_Dataset/Direct Observations.zip!/Direct Observations/190615_A_SO_AS_1_DO.pdf]

### 1.1.1 Instrument F –Direct Observation Form

| **<<Instrument F Direct Observation Form>>** |
|---|
| **PI Name:** PragyaMathema,Stephen Kodish<br>**Study Title:** Formative research to develop an infant and young child feeding (IYCF) intervention with micronutrient powder (MNP) for children aged 6 – 23 months<br>**PI Version/Date:** Version1.0/January 24, 2015 |

Date: ___19TH JUNE, 2015_________________Location: _____SONG L.G.A________________________

Data Collector: ___ANTIYA SANDA___________________Participant ID: _A HOUSE BY THE RIVER SIDE___

Main goals of this direct observation are to answer the following questions:

1. In this household, what would be the best way(s) to give nutritional supplements to young children? Why?

2. In this household, describe the food sharing that you observe throughout the meal. Are there any predictable patterns?

3. In this household, who is favored and how is this favoritism expressed during meal preparation and eating?

(TEAR OFF AT PERFORATED DOTTED LINE BELOW)

------------------------------------------------------------------------------------------------------------------------

PARTICIPANT ID: _________

Household Composition Table (Fill out as you do data collection)

| INITIALS | SEX (M/F) | AGE (estimated) | Relationship to household | Description |
|---|---|---|---|---|
| SB | F | 29 YEARS | MOTHER | A SLIM BLACK WOMAN |
| TB | M | 35 YEARS | FATHER | A TALL BLACK MAN |
| FB | F | 6 MONTHS | CHILD | A FAIR LITTLE BABY GIRL |
| BW | F | 4 YEARS | CHILD | A GIRL CHILD WITH A BLUE WRAPPER |
| SW | M | 25 YEARS | RELATIVE | A TALL SLIM MAN WITH A BLUE TROUSER AND WHITE SHIRT |
| | | | | |

| | | | | |
|---|---|---|---|---|
| | | | | |
| | | | | |
| | | | | |
| | | | | |
| | | | | |
| | | | | |
| | | | | |
| | | | | |

PARTICIPANT ID: __________

| TIME | ACTOR INITIALS | RECIPIENT INITIALS | WHAT HAPPENED | FOOD | Reactivity |
|---|---|---|---|---|---|
| 8:00 | SB | | SWEEPING | | |
| 8:05 | SB | | SWEEPING | | |
| 8:10 | SB | | SWEEPING | | |
| 8:15 | SB | | WASHING THE PLATES | | |
| 8:20 | BW | FB | CARRYING HER SISTER ON THE LAP | | |
| 8:25 | SB | | WASHING THE PLATES | | |
| 8:30 | SB | | WASHING THE PLATES | | |
| 8:35 | FB | | SITTING ON THE MAT | | |
| 8:40 | SB | | BRINGING OUT DIRTY CLOTHES | | |
| 8:45 | SB | | WASHING DIRTY CLOTHES | | |

| TIME | ACTOR INITIALS | RECIPIENT INITIALS | WHAT HAPPENED | FOOD | Reactivity |
|---|---|---|---|---|---|
| 8:50 | SB | | FETCHES WATER | | |
| 8:55 | SB | | WASHING CLOTHES | | |
| 9:00 | SB | | STILL WASHING | | |
| 9:05 | SB | FB | BREAST FEEDING HER BABY | | |
| 9:10 | SB | FB | BREAST FEEDING | | |
| 9:15 | SB | FB | STILL BREAST FEEDING | | |
| 9:20 | SB | FB | BREAST FEEDING | | |
| 9:30 | SB | FB | BREAST FEEDING | | |
| 9:35 | SB | | KEEPS HER BABY ON THE MAT | | |
| 9:40 | SB | | CONTINUED WASHING CLOTHES | | |
| 9:45 | FB | | SITTING AND PLAYING ON THE MAT | | |
| 9:50 | SB | | WASHING CLOTHES | | |
| 9:55 | SB | | RINSING CLOTHES | | |
| 10:00 | SB | | STILL RINSING CLOTHES | | |
| 10:05 | SB | | RINSING THE CLOTHES | | |
| 10:10 | FB | | STARTS CRYING | | |
| 10:15 | SB | | DRYING THE CLOTHES ON THE LINE | | |
| 10:20 | BW, SB | | PLAYING TOGETHER | | |
| 10:25 | SB | | STARTS PEELING GROUNDNUT | GROUNDNUT | |
| 10:30 | SB | | PEELING GROUNDNUTS | GROUNDNUT | |
| 10:35 | SB | | PEELING GROUNDNUTS | GROUNDNUT | |

| TIME | ACTOR INITIALS | RECIPIENT INITIALS | WHAT HAPPENED | FOOD | Reactivity |
|---|---|---|---|---|---|
| | | | | | |
| 10:40 | SB | | PEELING GROUNDNUTS | GROUNDNUT | |
| 10:45 | SB | | PEELING GROUNDNUTS | GROUNDNUT | |
| 10:50 | SB | | PEELING GROUNDNUTS | GROUNDNUT | |
| 10:55 | SB | | PEELING GROUNDNUTS | GROUNDNUT | |
| 11:00 | FB | | START CRYING | | |
| 11:05 | SB | | STARTS ARRANGING THE PLATES | | |
| 11:10 | FB | | SITTED QUIETLY ON THE MAT | | |
| 11:15 | SB | | WASHING A CUP | | |
| 11:20 | SB | | START MAKING FIRE | | |
| 11:21 | SB | | RINSING A POT | | |
| 11:22 | SB | | POURS WATER INTO POT | | |
| 11:23 | SB | | BLOWS THE FIRE | | |
| 11:24 | SB | | PUTS POT ON THE FIRE | | |
| 11:25 | SB | | PICKING OUT STONE FROM BEANS | | |
| 11:26 | SB | | STILL PICKING BEANS | | |
| 11:27 | SB | | PICKING BEANS | | |
| 11:28 | SB | | BRINGS OUT GROUNDNUT PASTE | GROUNDNUT | |
| 11:29 | SB | | POURS MORE WATER INTO POT ON FIRE | WATER | |
| 11:30 | SB | | MIXES GROUNDNUT PASTE WITH WATER | GROUNDNUT AND WATER | |
| 11:31 | SB | | MIXES GROUNDNUT PASTE WITH WATER | GROUNDNUT AND WATER | |

| TIME | ACTOR INITIALS | RECIPIENT INITIALS | WHAT HAPPENED | FOOD | Reactivity |
|---|---|---|---|---|---|
| 11:32 | SB | | BRINGS ANOTHER PLATE | | |
| 11:33 | SB | | BRINGS A BOWL | | |
| 11:34 | SB | | BEGINS TO SIEVE THE GROUNDNUT PASTE MIXED WITH WATER | GROUNDNUT PASTE AND WATER | |
| 11:35 | SB | | CONTINUED TO SIEVE THE GROUNDNUT PASTE | GROUNDNUT PASTE | |
| 11:36 | SB | | SIEVING GUINEA CORN FLOUR | GUINEA CORN | |
| 11:37 | SB | | MIXES GUINEA CORN FLOUR WITH WATER | GUINEA CORN | |
| 11:38 | SB | | POURS GUINEA CORN INTO THE POT ON THE FIRE | GUINEA CORN | |
| 11:39 | SB | | POURS GROUNDNUT INTO THE POT | GROUNDNUT | |
| 11:40 | SB | | STIRS THE GROUNDNUT AND GUINEA CORN | GROUNDNUT AND GUINEA CORN | |
| 11:45 | SB | | FETCHES SOME KUNU (PAP) INTO A BOWL | GUINEA CORN AND GROUNDNUT | |
| 11:46 | SB | | POURS A LITTLE KUNU INTO THE BOWL | GUINEA CORN AND GROUNDNUT | |
| 11:47 | SB | | BLOWS AIR INTO THE KUNU TO MAKE IT COLD | GUINEA CORN AND GROUNDNUT | |
| 11:48 | SB | | STILL BLOWING THE KUNU | GUINEA CORN AND GROUNDNUT | |
| 11:49 | SB | | BRINGS ANOTHER CUP | | |
| 11:50 | SB | | BRINGS A SPOON | | |
| 11:51 | SB | | FETCHES THE KUNU INTO A CUP | GUINEA CORN AND GROUNDNUT | |
| 11:52 | SB | | BROUGHT MNP | MNP | |

| TIME | ACTOR INITIALS | RECIPIENT INITIALS | WHAT HAPPENED | FOOD | Reactivity |
|---|---|---|---|---|---|
| 11:53 | SB | | UNSEAL MNP | MNP | |
| 11:54 | SB | | POURS MNP INTO KUNU | GUINEA CORN, GROUNDNUT AND MNP | |
| 11:55 | SB | | STIRS THE MNP WITH THE KUNU | GUINEA CORN, GROUNDNUT AND MNP | |
| 11:56 | SB | | DROPS THE CUP CONTAINING THE KUNU | | |
| 11:57 | SB | FB | CARRYS HER BABY | | |
| 11:58 | SB | | CARRY HER BABY ON THE LAP | | |
| 11:59 | SB | | STARTED FEEDING HER BABY WITH THE KUNU | GUINUE CORN, GROUNDNUT AND MNP | |
| 12:00 | SB | FB | CONTINUED TO FEED HER CHILD | GUINUE CORN, GROUNDNUT AND MNP | |
| 12:01 | SB | FB | FEEDING HER BABY WITH THE KUNU | GUINUE CORN, GROUNDNUT AND MNP | |
| 12:02 | SB | FB | FEEDING HER BABY WITH THE KUNU | GUINUE CORN, GROUNDNUT AND MNP | |
| 12:03 | SB | FB | FEEDING HER BABY SLOWLY | GUINUE CORN, GROUNDNUT AND MNP | |
| 12:05 | SB | FB | FEEDING HER BABY | GUINUE CORN, GROUNDNUT AND MNP | |
| 12:10 | SB | | KEEPS HER CHILD ON THE MAT | | |

| TIME | ACTOR INITIALS | RECIPIENT INITIALS | WHAT HAPPENED | FOOD | Reactivity |
|---|---|---|---|---|---|
| | | | | | |
| 12:15 | SB | | TAKES THE CUP FOR WASHING | | |
| 12:20 | SB | | CLEANS THE BABY'S BODY WITH TOWEL | | |
| 12:25 | SB | | REMOVES THE CHILD'S CLOTHES | | |
| 12:30 | SB | | CARRY THE CHILD FROM THE MAT | | |
| 12:35 | SB | | FETCHES WATER FROM A BIG BOWL | WATER | |
| 12:40 | SB | | IS BATHING HER CHILD | | |
| 12:45 | SB | | BATHING HER CHILD | | |
| 12:50 | SB | | BRINGS TOWEL TO CLEAN HER BABY'S BODY | | |
| 12:55 | SB | | DRESS UP HER CHILD | | |
| 1:00 | SB | FB | STATRTS BREASTFEEDING HER CHILD | | |
| 1:05 | SB | FB | BREASTFEEDING | | |
| 1:10 | SB | FB | BREASTFEEDING | | |
| 1:15 | SB | FB | STILL BREASTFEEDING | | |
| 1:20 | SB | FB | BREASTFEEDING | | |
| 1:25 | SB | FB | BREASTFEEDING | | |
| 1:30 | FB | | STARTS SLEEPING | | |
| 1:35 | FB | | SLEEPING | | |
| 1:40 | FB | | SLEEPING | | |
| 1:45 | FB | | SLEEPING | | |
| 1:50 | FB | | SLEEPING | | |

| TIME | ACTOR INITIALS | RECIPIENT INITIALS | WHAT HAPPENED | FOOD | Reactivity |
|---|---|---|---|---|---|
| 1:55 | FB | | SLEEPING | | |
| 2:00 | SB | | LAID HER CHILD ON THE MAT | | |
| 2:01 | SB | | BRINGS A BOWL | | |
| 2:02 | SB | | BRINGS A SIEVE | | |
| 2:03 | SB | | STARTS SIEVING MAIZE FLOUR | MAIZE | |
| 2:04 | SB | | STILL SIEVING MAIZE | MAIZE | |
| 2:05 | SB | | SIEVING MAIZE FLOUR | MAIZE | |
| 2:06 | SB | | SIEVING MAIZE FLOUR | MAIZE | |
| 2:07 | SB | | SIEVING THE MAIZE FLOUR | MAIZE | |
| 2:08 | SB | | SIEVING THE MAIZE FLOUR | MAIZE | |
| 2:09 | SB | | SIEVING THE MAIZE FLOUR | MAIZE | |
| 2:10 | SB | | SIEVING THE MAIZE FLOUR | MAIZE | |
| 2:11 | SB | | SIEVING THE MAIZE FLOUR | MAIZE | |
| 2:12 | SB | | SIEVING THE MAIZE FLOUR | MAIZE | |
| 2:13 | SB | | PUTS BIG POT ON FIRE | MAIZE | |
| 2:14 | SB | | POURED WATER INTO THE POT | WATER | |
| 2:15 | SB | | COVERED THE POT | | |
| 2:16 | SB | | BRINGS A BIG CALABASH | | |
| 2:17 | SB | | FETCHES SOME MAIZE INTO CALABASH | MAIZE | |
| 2:18 | SB | | POURS WATER INTO MAIZE FLOUR | MAIZE AND WATER | |
| 2:19 | SB | | MIXED THE MAIZE WITH WATER | MAIZE AND WATER | |
| 2:20 | SB | | POURED MIXED MAIZE WITH WATER INTO BOILING WATER ON THE FIRE | MAIZE AND WATER | |
| 2:21 | SB | | STIRING THE MAIZE IN BOILING WATER | MAIZE | |
| 2:22 | SB | | STIRING THE MAIZE WHILE COOKING | MAIZE | |
| 2:23 | SB | | STILL STIRING THE MAIZE | MAIZE | |
| 2:24 | SB | | STILL STIRING THE MAIZE | MAIZE | |
| 2:25 | SB | | STILL STIRING THE MAIZE | MAIZE | |
| 2:30 | SB | | COVERED THE POT ON FIRE | MAIZE | |
| 2:35 | SB | | SITTING ON THE MAT | | |
| 2:40 | SB | | SITTING AND WATCHING THE FIRE | | |

| TIME | ACTOR INITIALS | RECIPIENT INITIALS | WHAT HAPPENED | FOOD | Reactivity |
|---|---|---|---|---|---|
| 2:45 | SB | | BLOWS THE FIRE WHILE SITTING | | |
| 2:46 | SB | | POURED MAIZE FLOUR INTO THE POT WHILE COOKING | MAIZE | |
| 2:47 | SB | | STIRING THE FOOD | MAIZE | |
| 2:48 | SB | | STILL STIRING THE FOOD WITH A STICK | MAIZE | |
| 2:49 | SB | | CONTINUED STIRING THE FOOD ON FIRE | MAIZE | |
| 2:50 | SB | | CONTINUED STIRING | MAIZE | |
| 2:51 | SB | | REMOVE THE STICK AND COVERED THE POT | | |
| 2:52 | SB | | BLOWS THE FIRE | | |
| 2:53 | SB | | USING A PLATE TO BLOW THE FIRE | | |
| 2:54 | SB | | DROPPED THE PLATE | | |
| 2:55 | SB | | SAT ON THE MAT | | |
| 3:00 | SB | | SITTING AND WATCHING THE FIRE | | |
| 3:01 | SB | | BROUGHT DIFFERENT PLATES | | |
| 3:02 | SB | | SERVING TUWO (MAIZE PUDDING) INTO THE PLATES | MAIZE | |
| 3:03 | SB | | SERVING TUWO | MAIZE | |
| 3:04 | SB | | SERVING TUWO | MAIZE | |
| 3:05 | SB | | COVERS EACH PLATE | | |
| 3:10 | SB | | GOES TO THE ROOM TO BRING HER CHILD | | |
| 3:15 | FB | | WAKES UP FROM SLEEP | | |
| 3:20 | FB | | STARTS CRYING | | |
| 3:25 | SB | FB | BREASTFEEDING | | |
| 3:30 | SB | FB | BREASTFEEDING | | |
| 3:35 | SB | FB | BREASTFEEDING | | |
| 3:40 | SB | FB | BREASTFEEDING | | |
| 3:45 | SB | FB | BREASTFEEDING | | |
| 3:50 | SB | FB | BREASTFEEDING | | |
| 3:55 | SB | | KEPT HER CHILD ON THE MAT | | |
| 4:00 | SB | | STARTS SWEEPING THE COMPUND | | |
| 4:05 | SB | | CONTINUE TO SWEEP | | |
| 4:10 | FB | | IS PLAYING ON THE MAT | | |
| 4:15 | SB, FB | | SITTING TOGETHER ON THE MAT | | |
| 4:20 | SB, FB | | SITTING TOGETHER ON THE MAT | | |

| TIME | ACTOR INITIALS | RECIPIENT INITIALS | WHAT HAPPENED | FOOD | Reactivity |
|---|---|---|---|---|---|
| 4:25 | TB | | WALKED IN AND GOES TO THE ROOM | | |
| 4:30 | SB, FB | | CARRIED THE BABY AND SAT | | |
| 4:35 | SB | | ENTERED THE ROOM TO MEET HER HUSBAND | | |
| 4:40 | FB, SW | | SITTING AND PLAYING | | |
| 4:45 | FB, SW | | PLAYING TOGETHER | | |
| 5:00 | SB | FB | BREASTFEEDING | | |
| 5:05 | SB | FB | BREASTFEEDING | | |
| 5:10 | SB | FB | BREASTFEEDING | | |
| 5:15 | SB | FB | BREASTFEEDING | | |
| 5:20 | TB, FB | | CARRIED HIS BABY WHILE SITTING | | |
| 5:25 | TB, FB | | HOLDING HIS BABY ON HIS ARMS | | |
| 5:30 | TB, FB | | PLAYING WITH THE BABY | | |
| 5:35 | SB | | STARTS TO FETCH WATER | WATER | |
| 5:40 | SB | | FETCHING WATER INTO A BIG POT | WATER | |
| 5:45 | BW, SB | | PLAYING TOGETHER ON THE MAT | | |
| 5:46 | SB | | BROUGHT A POT OF SOUP FROM THE ROOM | BOUBAB SOUP | |
| 5:47 | SB | | WARMING THE POT OF SOUP | BOUBAB SOUP | |
| 5:48 | SB | | OPENED THE POT AND STIRED THE SOUP | BOUBAB SOUP | |
| 5:49 | SB | | COVERED THE POT | | |
| 5:50 | SB | | BROUGHT SOUP DISHES | | |
| 5:51 | SB | | SERVED SOUP INTO THE DISHES | BOUBAB SOUP | |
| 5:52 | SB | | SERVING THE BOUBAB SOUP | BOUBAB SOUP | |
| 5:52 | SB | | SERVING THE BOUBAB SOUP | BOUBAB SOUP | |
| 5:53 | SB | | COVERED EACH SOUP BOWL | BOUBAB SOUP | |
| 5:54 | SB | | CARRIED THE PLATE TO THE KITCHEN | | |
| 5:55 | SB | | PUT OFF THE FIRE ON THE WOOD | | |
| 5:56 | SB | | PUT OFF THE FIRE ON THE WOOD | | |
| 5:57 | SB | | CARRIED THE POT OF SOUP TO THE KITCHEN | | |

| TIME | ACTOR INITIALS | RECIPIENT INITIALS | WHAT HAPPENED | FOOD | Reactivity |
|---|---|---|---|---|---|
| 5:58 | SB | | COLLECTS THE BABY FROM THE FATHER | | |
| 5:59 | SB | | STARTS BREASTFEEDING | | |
| 6:00 | SB | | BREASTFEEDING | | |

| | | | | | |
|---|---|---|---|---|---|
| | | | | | |
| | | | | | |
| | | | | | |
| | | | | | |
| | | | | | |
| | | | | | |
| | | | | | |
| | | | | | |
| | | | | | |
| | | | | | |
| | | | | | |
| | | | | | |
| | | | | | |
| | | | | | |
| | | | | | |
| | | | | | |
| | | | | | |

| | | | | | |
|---|---|---|---|---|---|
| | | | | | |
| | | | | | |
| | | | | | |
| | | | | | |
| | | | | | |
| | | | | | |
| | | | | | |
| | | | | | |
| | | | | | |
| | | | | | |
| | | | | | |
| | | | | | |
| | | | | | |
| | | | | | |
| | | | | | |
| | | | | | |
| | | | | | |
| | | | | | |
| | | | | | |
| | | | | | |
| | | | | | |

| | | | | | |
|---|---|---|---|---|---|
| | | | | | |
| | | | | | |
| | | | | | |
| | | | | | |
| | | | | | |
| | | | | | |
| | | | | | |
| | | | | | |
| | | | | | |
| | | | | | |
| | | | | | |
| | | | | | |
| | | | | | |
| | | | | | |
| | | | | | |
| | | | | | |
| | | | | | |
| | | | | | |
| | | | | | |
| | | | | | |
| | | | | | |

| | | | | | |
|---|---|---|---|---|---|
| | | | | | |
| | | | | | |
| | | | | | |
| | | | | | |
| | | | | | |
| | | | | | |
| | | | | | |
| | | | | | |
| | | | | | |
| | | | | | |
| | | | | | |
| | | | | | |
| | | | | | |
| | | | | | |
| | | | | | |
| | | | | | |
| | | | | | |
| | | | | | |
| | | | | | |
| | | | | | |
| | | | | | |
| | | | | | |

| | | | | | |
|---|---|---|---|---|---|
| | | | | | |
| | | | | | |
| | | | | | |
| | | | | | |
| | | | | | |
| | | | | | |
| | | | | | |
| | | | | | |
| | | | | | |
| | | | | | |
| | | | | | |
| | | | | | |
| | | | | | |
| | | | | | |
| | | | | | |
| | | | | | |
| | | | | | |
| | | | | | |
| | | | | | |
| | | | | | |
| | | | | | |

| | | | | | |
|---|---|---|---|---|---|
| | | | | | |
| | | | | | |
| | | | | | |
| | | | | | |
| | | | | | |
| | | | | | |
| | | | | | |
| | | | | | |
| | | | | | |
| | | | | | |
| | | | | | |
| | | | | | |
| | | | | | |
| | | | | | |
| | | | | | |
| | | | | | |
| | | | | | |
| | | | | | |
| | | | | | |
| | | | | | |
| | | | | | |

| | | | | | |
|---|---|---|---|---|---|
| | | | | | |
| | | | | | |
| | | | | | |
| | | | | | |
| | | | | | |
| | | | | | |
| | | | | | |
| | | | | | |
| | | | | | |
| | | | | | |
| | | | | | |
| | | | | | |
| | | | | | |
| | | | | | |
| | | | | | |
| | | | | | |
| | | | | | |
| | | | | | |
| | | | | | |
| | | | | | |
| | | | | | |

| | | | | | |
|---|---|---|---|---|---|
| | | | | | |
| | | | | | |
| | | | | | |
| | | | | | |
| | | | | | |
| | | | | | |
| | | | | | |
| | | | | | |
| | | | | | |
| | | | | | |
| | | | | | |
| | | | | | |
| | | | | | |
| | | | | | |
| | | | | | |
| | | | | | |
| | | | | | |
| | | | | | |
| | | | | | |
| | | | | | |
| | | | | | |
| | | | | | |

| | | | | | |
|---|---|---|---|---|---|
| | | | | | |
| | | | | | |
| | | | | | |
| | | | | | |
| | | | | | |
| | | | | | |
| | | | | | |
| | | | | | |
| | | | | | |
| | | | | | |
| | | | | | |
| | | | | | |
| | | | | | |
| | | | | | |
| | | | | | |
| | | | | | |
| | | | | | |
| | | | | | |
| | | | | | |
| | | | | | |
| | | | | | |

| | | | | | |
|---|---|---|---|---|---|
| | | | | | |
| | | | | | |
| | | | | | |
| | | | | | |
| | | | | | |
| | | | | | |
| | | | | | |
| | | | | | |
| | | | | | |
| | | | | | |
| | | | | | |
| | | | | | |
| | | | | | |
| | | | | | |
| | | | | | |
| | | | | | |
| | | | | | |
| | | | | | |
| | | | | | |
| | | | | | |
| | | | | | |

| | | | | | |
|---|---|---|---|---|---|
| | | | | | |
| | | | | | |
| | | | | | |
| | | | | | |
| | | | | | |
| | | | | | |
| | | | | | |
| | | | | | |
| | | | | | |
| | | | | | |
| | | | | | |
| | | | | | |
| | | | | | |
| | | | | | |
| | | | | | |
| | | | | | |
| | | | | | |
| | | | | | |
| | | | | | |
| | | | | | |
| | | | | | |

| | | | | | |
|---|---|---|---|---|---|
| | | | | | |

PARTICIPANT ID: _________

Primary Questions (Complete after you complete data collection):

From what you have seen observing THIS HOUSEHOLD, please answer the following questions in detail:

| Question | Write your detailed response here |
|---|---|
| 1. In this household, what would be the best way(s) to give nutritional supplements such as an MNP to young children to ensure compliance?<br><br>Why? | THE BEST WAY TO GIVE MNP TO THE YOUNG CHILD IS TO MIX THE MNP WITH THE CHILDS KUNU (PAP) EVERY MORNING (THE YOUNG CHILDREN DRINKS EITHER GUINEA CORN OR MILLET PAP EVERY MORNING).<br><br>BECAUSE WHEN THE MNP IS MIXED IN KUNU, IT DOESN'T LOOK DIFFERENT. THEREFORE, THE CHILD WILL EASILY AGREE TO DRINK. |
| 2. In this household, who is favored during meal preparation or feeding?<br><br>How is this favoritism expressed? | THE YOUNGEST CHILD OF 6 MONTHS IS FAVOURED ABOVE ALL OTHER CHILDREN.<br><br>IT IS EXPRESSED IN SUCH A WAY THAT, THE YOUNGEST CHILD'S KUNU IS PREPARED ALONE, BEFORE THE OTHER CHILDRENS KUNU IS PREPARED AFTERWARDS. |
| 3. How could we promote the MNP supplement for this family that would attract | THE MNP SHOULD BE PROMOTED THROUGH THE DAD, BECAUSE HE IS AWARE OF EVERY THING THAT GOES ON AND AROUND THE |

| | |
|---|---|
| their attention?<br><br>Who would they listen to? | HOUSE.<br><br>THEY WOUD LISTEN TO THEIR FATHER MORE BECAUSE HE IS THE HEAD OF THE HOUSEHOLD AND ALL THE CHILDREN ARE SCARED OF HIM. SCARED IN THE SENSE THAT, IF HE GIVES ANY INSTRUCTION ON WHAT NEEDS TO BE DONE, IT IS DONE OMMEDIATELY WITHOUT ANY DELAY. |
| 4. In this household, what would be the best ways to promote the MNP but limit how much it would be shared?<br><br>Why? | THE MNP SHOULD BE PROMOTED THROUGH MOTHERS ALSO SINCE SHE STAYS ALL THROUGH THE DAY WITH THE CHILD. SINCE THE YOUNGEST CHILD IS 6 MONTH OLD AND HAS JUST LEARNED HOW TO EAT, THE MNP CANNOT BE SHARED.<br><br>THE REASON IS BECAUSE THE YOUNGEST CHILD'S FOOD IS PREPARED SEPARETELY AND ONLY THE YOUNGEST CHILD'S KUNU GETS THE MNP. THE CHANCES THAT MNP WILL BE SHARED IS VERY LIMITED. |
| 5. What other issues and concerns did you see with this family that are important | IGNORANCE ON THE PART OF THE MOTHER ON WHAT TO ADD IN PREPARING THE KUNU AND THE HYGIENE TO MAINTAIN IN |

| | |
|---|---|
| for us to consider with regard to introduction of an MNP?<br><br>Why? | PREPARING THE KUNU IS VERY POOR.<br><br>THE IDEAL WAY TO PREPARE KUNU IS TO ADD GROUNDNUT PASTE INTO EITHER THE GUINEA CORN FLOUR OR MAIZE FLOUR. THE MOTHER DOES NOT KNOW THAT GROUNDNUT IS NECESSARY UNTIL I TOLD HER SO AND WHEN SHE STARTS PREPARING THE KUNU, FLIES WERE PERCHING ALL OVER THE KUNU |
| 6. Based on your experience observing this child today, do you believe reactivity negatively impacted your ability to see the food-related behaviors of the child?<br><br>Please explain your answer in detail. | NOT IN ANY WAY<br><br>THERE WAS NO REACTIVITY |
